# A Role for Neural Modeling in the Study of Brain Disorders

**DOI:** 10.3389/fnsys.2012.00057

**Published:** 2012-08-06

**Authors:** Barry Horwitz, Arpan Banerjee

**Affiliations:** ^1^Brain Imaging and Modeling Section, National Institute on Deafness and Other Communication Disorders, National Institutes of HealthBethesda, MD, USA

In the past few decades, clinical research on brain disorders has become enriched by the addition of a number of new tools for characterizing and assessing brain function. Before the 1960s, clinical examination, behavioral observation (including batteries of neuropsychological tests), and electroencephalography (EEG) were the primary research tools. Since then, new additions to the repertoire have included genetic analysis, and importantly for our purposes, brain imaging (both structural and functional). Furthermore, the amount of data that can be acquired by the genetic and neuroimaging tools has grown to such levels that advanced and intensive computational methods are required to analyze the data. However, computational techniques can be used for more than data analysis, as a recent paper in this issue by Wendling et al. (2010) demonstrates. In an article that reviews a variety of computational methods for characterizing functional brain connectivity from electrophysiological data, specifically in the context of partial epilepsy, Wendling et al. also employ a physiologically plausible neural model to generate simulated data and illustrate how such modeling can be used to help interpret the experimental data. This Wendling et al. study, along with some others (Horwitz et al., 1995; Schlosser et al., 2006; Husain, 2007; Alstott et al., 2009; Kim and Horwitz, 2009; van Albada et al., 2009; Rowe, 2010; Seghier et al., 2010), suggests that neural modeling may soon become a useful addition to the clinical research toolkit for investigating brain disorders.

The particular issue addressed by Wendling et al. (2010) centers on identifying, prior to brain surgery, the epileptogenic zone (EZ) responsible for seizures in patients with partial epilepsy. The data come from depth electrode EEG recordings obtained during pre-surgical evaluation. What makes such data hard to interpret is that the spatial organization of the EZ corresponds to a network of distributed neural populations “showing ‘hyperexcitability’ and ‘hypersynchronization’ properties” (Wendling et al., 2010). In an attempt to unravel the relationships between these various populations, a variety of signal processing techniques have been proposed to determine the functional connectivities among the epileptogenic network neural populations. Wendling et al. illustrate one such functional connectivity method in detail, non-linear regression analysis.

However, the availability of large numbers of potential methods for evaluating the statistical relationships between time-varying signals, each resting on specific assumptions about the underlying neural relationship between the signals, makes it imperative to have a way to see whether a given method actually produces a result that properly reflects the underlying neural relationships (for a recent review, see Banerjee et al., 2012a). For this purpose, Wendling et al. (2010) demonstrate how a biologically realistic model of coupled neuronal populations can be employed to simulate EEG data similar to the data that are experimentally acquired. They apply the non-linear regression analysis method to the simulated data and show that this particular connectivity method captures the “ground truth” of the relationships embedded in the model. Because in an actual brain from which data are collected we have no way of knowing the relationships among the network elements, this use of neural modeling thus allows one to partially verify that a functional connectivity method permits an interpretation that corresponds to the underlying relationship between the neural elements.

Using neural modeling to help assess the interpretability of various functional and effective brain connectivity methods has started to become an important component for analyzing connectivity analysis techniques. Examples of previous studies that utilized this approach include Kim and Horwitz (2009), Wendling et al. (2009), and Banerjee et al. (2012b). However, there are other uses by which neural modeling can play a significant role in the clinical research of brain disorders. One type of neural modeling, what has been called systems-level neural modeling (Horwitz et al., 1999), attempts to evaluate the strengths of the direct connections between brain regions (i.e., the interregional effective connectivity), and has been applied to functional neuroimaging data in a number of studies to assess differences between patients and healthy subjects (e.g., Horwitz et al., 1995; Schlosser et al., 2006; Rowe, 2010; Seghier et al., 2010). This approach takes the acquired data and utilizes it to fit the parameters of the model. However, one can turn the approach around and simulate data (at the brain region level) and explore the simulated data to determine how parameter modification that may characterize a brain disorder alters the simulated neuroimaging signals. A good example of this can be found in Alstott et al. (2009), where the impact of brain lesions on simulated resting fMRI data was assessed.

A third way in which neural modeling is beginning to contribute as a clinical neuroscience tool is in terms of helping understand the neural basis of a disorder. Here, one would use a large-scale neural model consisting of multiple neuronal units in several brain regions. Specific proposals concerning the neural substrate of a disorder could be incorporated into the neural model by explicit changes in model parameter values; simulated data could then be generated and compared with corresponding experimental data. An fMRI example is found in Husain’s (2007) work on tinnitus and an EEG illustration comes from a study by van Albada et al. (2009) related to Parkinson's disease.

Obviously, neural modeling, like any other method, has limitations. For example, an important simplification of the class of neural models used by Wendling et al. (2010) arises from the use of a mean-field assumption: the activity of a brain region is represented by the summed membrane potentials rather than neural spiking activity. Computational work is currently in progress to relate the more realistic neural spiking approach to the mean-field modeling framework (Coombes, 2010). Overcoming such limitations should result in greater use of neural modeling as a useful clinical research tool.
